# Collagen-Type Composition in the Semitendinosus, Quadriceps, and Patellar Tendons of a 22-Year-Old Patient: A Case Report

**DOI:** 10.7759/cureus.61324

**Published:** 2024-05-29

**Authors:** Yushin Mizuno, Yasushi Takata, Kazuaki Yoshioka, Satoru Demura, Junsuke Nakase

**Affiliations:** 1 Department of Orthopaedic Surgery, Graduate School of Medical Sciences, Kanazawa University, Kanazawa, JPN; 2 Section of Rehabilitation, Kanazawa University Hospital, Kanazawa, JPN; 3 Department of Physiology, Graduate School of Medical Sciences, Kanazawa University, Kanazawa, JPN

**Keywords:** collagen-type composition, semitendinosus tendon, quadriceps tendon, patellar tendon, graft failure, anterior cruciate ligament reconstruction

## Abstract

Graft failure is a common postoperative complication after anterior cruciate ligament (ACL) reconstruction. Recently, a theory has emerged that histological and microstructural factors of autografts may be related to graft failure. We simultaneously collected the semitendinosus tendon (ST), quadriceps tendon (QT), and patellar tendon (PT) from a 22-year-old patient to provide insights into the differences in the collagen-type composition of the three tendons in skeletally mature patients. These findings may serve as a basis for selecting autografts for ACL to reduce graft failure rates.

The patient was a 22-year-old female who required the removal of artificial ligament, screws, and washers and medial patellofemoral ligament (MPFL) reconstruction with an ST autograft after two surgeries for recurrent dislocation of the left patella. The ST, QT, and PT obtained during necessary intraoperative procedures were used as samples. The tissues were processed and immunostained; this was followed by confocal microscopy. Evaluation was performed by calculating the percentage of areas positive for collagen types I and III.The percentage of type I collagen in the ST, QT, and PT groups was 88%, 85%, and 88%, respectively.The collagen-type composition was examined following simultaneous collection of the ST, QT, and PT. The results revealed no significant differences in the content of physically strong type I collagen, which supports previous findings showing that the clinical outcomes after ACL reconstruction do not vary with the autograft used.

## Introduction

Reconstruction using the semitendinosus tendon (ST), quadriceps tendon (QT), or patellar tendon (PT) as an autograft is a common treatment for anterior cruciate ligament (ACL) injuries, which frequently occur in sports knee trauma [1]. Although a complete consensus has not yet been reached, there have been some reports of different postoperative clinical outcomes depending on the type of autograft used in ACL reconstruction [2]. Graft failure is the most serious postoperative complication following ACL reconstruction. Recently, the incidence of graft failure was reported to be particularly high after reconstruction using an ST autograft in a group of patients with immature physical growth before the closure of the epiphyseal line [3]. The cause of this problem was previously thought to be the unique background of adolescent patients (e.g., high activity level and low adherence to postoperative rehabilitation) [4]. However, a theory has recently emerged that histological and microstructural factors of autografts may be related to graft failure after ACL reconstruction. Moreover, it has been reported that the collagen fibril diameter and the microstructure of the ST and QT increase with age [5,6] and that the percentage of type III collagen, which is physically weak, is relatively high in the ST of patients with unclosed epiphyseal lines [7]. The collagen type in the skin is predominantly type III at birth, with the percentage of type I collagen increasing with age [8]. Similar changes may occur in the soft tissues of the tendons. Thus, the characteristics of autografts at the time of reconstruction are likely to vary with age and the degree of physical development. Type I collagen, the main component of the tendon, is rod-shaped and stiff, with high mechanical strength [9], whereas type III collagen, a secondary component of the tendon, is more flexible than type I collagen [10]. These differences in physical strength are related to the longevity of autografts and may contribute to the graft failure rate after ACL reconstruction.

We had the rare opportunity to simultaneously collect ST, QT, and PT data from a 22-year-old patient. We aimed to provide insights into the differences in the collagen-type composition of the ST, QT, and PT in skeletally mature patients. In conjunction with previous reports [7], our findings may provide a foundation for selecting autografts for ACL reconstruction to reduce graft failure rates. Before this case report, we hypothesized that the collagen-type composition of the ST, QT, and PT would be comparable in a skeletally mature 22-year-old female patient.

## Case presentation

This case report was approved by the Ethics Review Committee of our institution. The patient received oral and written information regarding the case report, and consent was obtained.

Patient information

**Figure 1:**
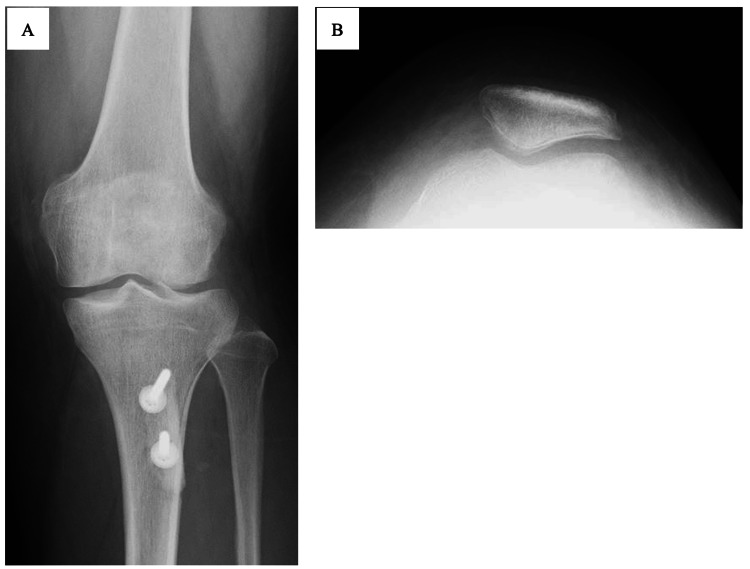
X-ray images of the 22-year-old female patient before hardware removal and medial patellofemoral ligament reconstruction Radiographic images of the left knee joint after tibial tuberosity osteotomy and medial patellofemoral ligament augmentation (A). The patella is in its normal position at 60° knee flexion (B).

The patient was a 22-year-old female who had undergone two surgical procedures (at seven years and four months, respectively, prior to presentation) for recurrent dislocation of the left patella. The first surgery involved medial patellofemoral ligament (MPFL) repair. However, the patella was re-dislocated in the subsequent course. Seven years later, the patient underwent tibial tuberosity osteotomy and MPFL augmentation using an artificial ligament (Figure 1). After the second surgery, the patient continued rehabilitation treatment; however, in addition to anterior knee pain, she had a limited range of motion of 80° in knee flexion. Magnetic resonance imaging (MRI) scan also showed cartilage damage at the femoral trochlea, suggesting that the tension of the artificial ligament used for the MPFL augmentation was too high, and the patient was referred to our hospital for additional treatment.

At the initial visit to our hospital, the bony alignment of the patient's left knee appeared to be slightly valgus, and the range of motion of the left knee was 0° in extension to 60° in flexion, a straight leg raise was possible, and the apprehension test was negative. On palpation, heat was felt on the tibial tuberosity. The range of motion of the right knee was 0° in extension to 145° in flexion, with the apprehension test being negative.

Surgical treatment and sampling

**Figure 2:**
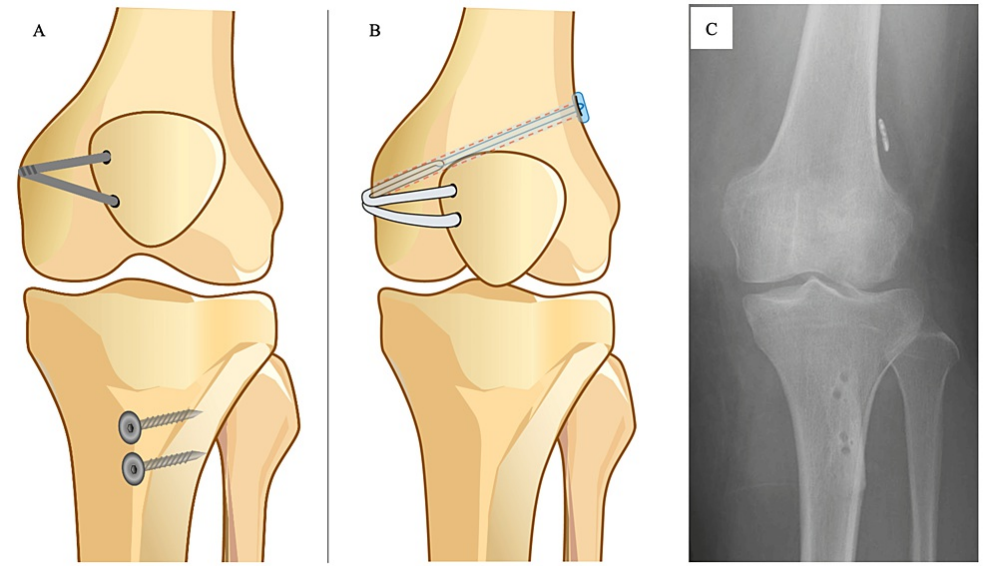
Preoperative and postoperative schemas and postoperative X-ray image The orthopedic surgeon first removed the two screws and two washers used for the tibial tuberosity osteotomy and the artificial ligament for medial patellofemoral ligament augmentation (A). Subsequently, medial patellofemoral ligament reconstruction was performed with the semitendinosus tendon (B). Postoperatively, the flexion contracture has improved (C).

Four months after the second surgery, a third surgery was performed at our hospital (Figure 2). Even under general anesthesia, the knee joint was flexed only up to 90°; therefore, the artificial ligament was removed on the patellar side, which enabled deep flexion. In addition, two screws and two washers used in the tibial tuberosity osteotomy were removed. Finally, we created a bone tunnel and performed MPFL reconstruction using the ST. The ST autograft was fixed at 60° of knee flexion, and the procedure was completed after confirming that the knee joint could be firmly and deeply flexed after fixation.

The ST, QT, and PT used in this case report were sampled during the procedure. Specifically, the ST can be obtained from the trimming process during the creation of the autograft. The QT and PT were obtained when part of the tendon attachment was detached, and the hardware was removed. No additional procedures were performed in this case report. The samples were considered unnecessary tissues that should have been removed.

Immunostaining and confocal microscopy

Tissue immunostaining was performed on 4% paraformaldehyde-fixed, paraffin-embedded tissue sections using a previously described standard protocol [11]. This method was also used in previous studies with similar content [7]. Briefly, the sections were deparaffinized and rehydrated in graded alcohol solutions. After antigen retrieval in DAKO Target Retrieval Solution (pH 9) (Agilent, Santa Clara, CA, USA) for 30 minutes, the sections were blocked with DAKO Protein Block (serum-free) (Agilent) and subjected to immunofluorescence staining using the following primary antibodies: goat polyclonal anti-collagen type I (goat IgG) (Southern Biotechnology, Birmingham, AL, USA) and mouse monoclonal anti-collagen type III (mouse IgG1) (clone FH-7A) (Abcam, Cambridge, UK) at 4°C overnight. After washing with 0.1% TritonX-100 in phosphate-buffered saline, the sections were treated with appropriate Alexa-Fluor 488/568-conjugated secondary antibodies (Molecular Probes; Thermo Fisher Scientific, Waltham, MA, USA) for one hour at room temperature (approximately 25°C), before mounting with 40,6-diamidino-2-phenylindole (DAPI) for nuclear staining. Confocal imaging was performed using an inverted Nikon Eclipse Ti2 confocal microscope (Nikon Instruments/Nikon Corp., Tokyo, Japan) equipped with an Andor Dragonfly spinning disk unit, Andor EMCCD camera (iXon DU888; Andor Technology Ltd., Oxford Instruments, Belfast, UK), and laser unit (Coherent Inc., Santa Clara, CA, USA). Excitation for the DAPI, Alexa-488, and 568 chromophores was provided by 405-nm, 488-nm, and 560-nm lasers, respectively.

Evaluation and results

**Figure 3:**
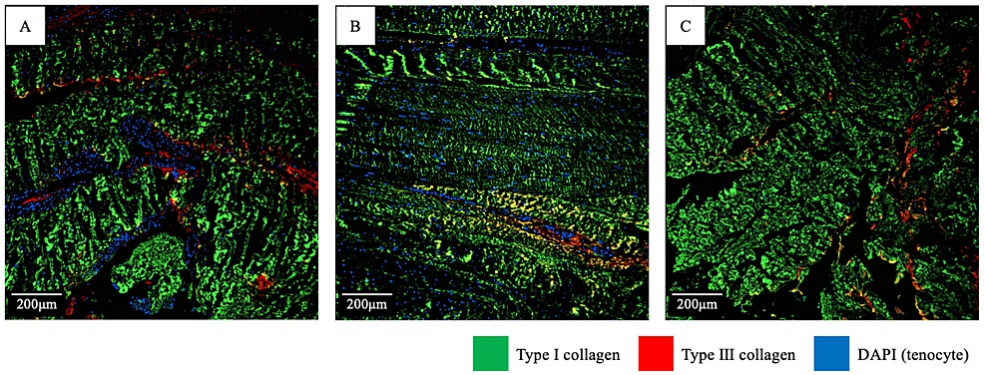
Immunostained images of the semitendinosus, quadriceps, and patellar tendons Immunostaining of the semitendinosus (a), quadriceps (b), and patellar (c) tendons to examine the collagen-type composition: the area contents of type I collagen are 88%, 85%, and 88%, respectively, with no significant difference. Green, red, and blue colors represent type I-, type III-, and DAPI (tenocyte)-positive areas, respectively; yellow areas indicate areas positive for both type I and III collagen. DAPI: 40,6-diamidino-2-phenylindole

The evaluation methods were similar to those used in a previous study [7]. To quantify type I and type III collagen-positive areas, images of 2-3 sagittal sections were quantitatively evaluated using Fiji/Image-J (https://fiji.sc). Images were converted into 8-bit Tagged Image File Format (TIFF) files, and the average stained areas for type I and type III collagen-positive regions from three random fields were calculated using the ImageJ Analyze Particle tool for each section. Areas of collagen positivity were manually thresholded by visual comparison of the images to ensure that the tool effectively resolved the anti-collagen antibody-stained lesions. Data were expressed as the percentage area of the collagen type per microscopic field. In other words, the sum of the positive percentages of type I and type III collagen in the evaluation results was 100%. Evaluations were performed in a blinded manner to avoid bias. Results showed that the percentages of type I collagen in the ST, QT, and PT were similar at 88%, 85%, and 88%, respectively (Figure 3). In other words, the percentages of physically weak type III collagen were 12%, 15%, and 12% in the ST, QT, and PT, respectively.

## Discussion

A valuable finding of this case report was the comparable percentages of collagen observed in the ST, QT, and PT obtained simultaneously from adult patients. This is the first report of a detailed case report of the collagen composition of the ST, QT, and PT in adult patients, which is made particularly rare by the simultaneous collection of samples. This finding may serve as a foundation for selecting autografts for ACL reconstruction to reduce graft failure rates.

First, the structural stability of collagen, the main component of the tendon, is related to its mechanical strength, which is affected by its intermolecular cross-linked structure [12]. In addition, the diameter of fibrils in the skin, which is the same soft tissue as that in tendons, tends to be larger for fibrils containing more type I collagen [13], and the larger the diameter of the collagen fibrils, the higher the mechanical strength [14,15]. In other words, the ST, QT, and PT from the 22-year-old skeletally mature patient evaluated in this case report had comparable collagen-type compositions, suggesting that these three tendon tissues have comparable physical strengths. Mizuno et al. [7] conducted a similar study using the ST, QT, and PT from a skeletally immature 11-year-old patient and reported that type III collagen in the ST accounted for approximately 40%, whereas most of the collagen in the QT and PT was type I. Taken together, these findings suggest that changes in the composition of tendon collagen type occur as the body and bones grow, with more pronounced changes in the ST. Regarding the evidence for this, the overall structure of collagen fibrils is strengthened by traction force [16]. It has also been reported that the orientation of collagen fibrils can be adjusted, and the density of collagen fibrils can be increased by traction, both of which occur rapidly in this context [17,18]. In summary, the mechanical strength of the ST in patients with complete bone growth was higher than that in patients with incomplete bone growth, and its histological composition may be comparable to that of the QT and PT. In support of this, the clinical and functional outcomes after ACL reconstruction with ST, QT, and PT autografts were comparable [1]. One explanation for this result may be that the percentage of type I collagen in pure ACL was 90% [19], which is similar to the composition of the ST, QT, and PT collagen types examined in this case report.

As explained in the Introduction, the findings of this case report are valuable. However, this case report also has a few limitations. First, the results did not consider the ligamentization process, which is a change that occurs in the autografts after ACL reconstruction and involves replacing a new ligament after reconstruction [13,20]. Therefore, the collagen composition of the autograft itself may change over time after surgery, and our results may not be applicable to the entire postoperative period. Second, the samples used in this case report were obtained from parts that were not used as autografts, and we were unable to evaluate the tendon parenchyma that served as the autograft. Both of these limitations are unavoidable from the standpoint of ethical considerations, and they are difficult to study using human-derived tissues. Third, no mechanical tests were performed, and the relationship between the collagen composition and mechanical strength of the samples used in this case report is essentially unknown. Unfortunately, the samples used in this case report were extremely small and not sufficiently large to allow for mechanical testing. However, previous reports have indirectly shown that the collagen-type composition of tendons is related to the collagen fibril diameter [13] and that the larger the collagen fibril diameter, the greater the mechanical strength [15].

As this was a case report of only one patient with recurrent patellar dislocation as the main symptom, the collagen composition of the patient's body may differ from that of healthy patients. However, the rarity of the evaluation of the ST, QT, and PT collected simultaneously has a high degree of creativity, which, together with the results of previous reports, represents novel findings. Further studies with larger numbers of cases will increase the credibility of this case report and lead to further developments in this field.

## Conclusions

The collagen-type composition was examined in the ST, QT, and PT samples collected simultaneously from a 22-year-old patient. The results revealed that the content of physically strong type I collagen was almost equal across the three samples, suggesting that the physical strength of autografts in adult females is comparable and may provide a foundation for selecting autografts for ACL to decrease graft failure rates.
